# Challenges in Stratifying the Molecular Variability of Patient-Derived Colon Tumor Xenografts

**DOI:** 10.1155/2018/2954208

**Published:** 2018-12-19

**Authors:** Magdalena Cybulska, Tomasz Olesinski, Krzysztof Goryca, Katarzyna Paczkowska, Malgorzata Statkiewicz, Michal Kopczynski, Aleksandra Grochowska, Katarzyna Unrug-Bielawska, Anita Tyl-Bielicka, Marta Gajewska, Andrzej Mroz, Michalina Dabrowska, Jakub Karczmarski, Agnieszka Paziewska, Leszek Zając, Mariusz Bednarczyk, Michal Mikula, Jerzy Ostrowski

**Affiliations:** ^1^Department of Genetics, Maria Sklodowska-Curie Institute–Oncology Centre, 02-781 Warsaw, Poland; ^2^Department of Gastroenterology, Hepatology and Clinical Oncology, Medical Center for Postgraduate Education, 01-813 Warsaw, Poland; ^3^Department of Gastroenterological Oncology, Maria Sklodowska-Curie Institute–Oncology Centre, 02-781 Warsaw, Poland

## Abstract

Colorectal cancer (CRC) is the second most common cancer in Europe and a leading cause of death worldwide. Patient-derived xenograft (PDX) models maintain complex intratumoral biology and heterogeneity and therefore remain the platform of choice for translational drug discovery. In this study, we implanted 37 primary CRC tumors and five CRC cell lines into NU/J mice to develop xenograft models. Primary tumors and established xenografts were histologically assessed and surveyed for genetic variants and gene expression using a panel of 409 cancer-related genes and RNA-seq, respectively. More than half of CRC tumors (20 out of 37, 54%) developed into a PDX. Histological assessment confirmed that PDX grading, stromal components, inflammation, and budding were consistent with those of the primary tumors. DNA sequencing identified an average of 0.14 variants per gene per sample. The percentage of mutated variants in PDXs increased with successive passages, indicating a decrease in clonal heterogeneity. Gene Ontology analyses of 4180 differentially expressed transcripts (adj. p value < 0.05) revealed overrepresentation of genes involved in cell division and catabolic processes among the transcripts upregulated in PDXs; downregulated transcripts were associated with GO terms related to extracellular matrix organization, immune responses, and angiogenesis. Neither a transcriptome-based consensus molecular subtype (CMS) classifier nor three other predictors reliably matched PDX molecular subtypes with those of the primary tumors. In sum, both genetic and transcriptomic profiles differed between donor tumors and PDXs, likely as a consequence of subclonal evolution at the early phase of xenograft development, making molecular stratification of PDXs challenging.

## 1. Introduction

Patient-derived xenografts (PDXs) are established by transferring tumor tissue from patients into immunosuppressed mice. After a period of dormancy, xenografts enter a logarithmic growth phase and may be reimplanted in subsequent generations of mice. PDXs are the most useful experimental models for predicting therapeutic responses and the final filters for selection of drug candidates for clinical trials and may also serve as an important source of new predictive biomarkers [1]. For these reasons, the US National Cancer Institute (NCI) is switching to PDX models from the NCI-60 panel of cell lines that have been used for nearly three decades in drug discovery [2]. To ascertain whether preclinical findings are translatable to clinical practice, PDXs must recapitulate the cellular and molecular characteristics of the donor tumors. As exemplified by a recently published study from the OncoTrack consortium [1], the use of PDXs in preclinical studies should be preceded by deep molecular evaluation of each PDX model.

Colorectal cancer (CRC) is the second most common cancer in Europe and a leading cause of death worldwide [3]. Although CRC mortality can be reduced by prevention and early detection, survival of patients with advanced disease depends on adjuvant therapies. Treatment strategies for CRC depend on cancer stage and location. For stage III colon cancer, wide surgical resection and anastomosis with a standard adjuvant chemotherapy (CHT) are routinely performed, whereas neoadjuvant radiochemotherapy is recommended for patients with locally advanced rectal cancer [4]. The combination of CHT with new targeted therapies, such as inhibitors of epidermal growth factor receptor (EGFR) and immunotherapies, further increases median survival [5]. However, therapeutic responses vary significantly due to primary and secondary mechanisms of resistance, which reflect inter- and intratumor heterogeneity. Although tumor heterogeneity can be measured based on detection of mutant allele frequencies within a given tumor and differences in cancer cell–intrinsic gene expression profiles, both approaches may be challenging, for example, due to loss of human immune signatures and stromal components or selection of preexisting cancer minor clones during PDX development and propagation [6–8].

Here, we compared the histological, genetic, and transcriptomic properties of PDXs and xenografts established from CRC cell lines (CLXs). Primary tumors, cell lines, and their corresponding P2 xenografts were surveyed for genetic variants and gene expression using a panel of 409 cancer-related genes and RNA sequencing (RNA-seq), respectively. Although most histological parameters remained stable between donor tumors and PDXs derived from them, both primary tumors and PDXs significantly differed from CLXs. In turn, both genetic and transcriptomic profiles differed between donor tumors and PDXs, likely as a consequence of subclonal evolution of PDXs at the early phase of xenograft development.

## 2. Materials and Methods

### 2.1. Xenograft Models

NU/J (nude) athymic mice were purchased from The Jackson Laboratory and maintained in a specific pathogen-free (SPF) facility. The core of the breeding colony consisted of a group of brother × sister mated animals kept in an internal bank of inbred strains. Mice intended for experiments were produced according to Lane–Petter's “traffic-light” system.

To establish PDXs, pieces (~10–20 mm^3^) of fresh colorectal carcinoma specimens, obtained after surgical resection, were implanted subcutaneously into both flanks of NU/J mice (passage 0, P0). Growth was monitored until the tumor reached a volume of about 1 cm^3^, at which point the tumor was excised and dissociated, and pieces (~10–20 mm^3^) were again implanted into a new set of mice (passage 1, P1). This procedure was repeated a second time. To establish a CLX, 1–5 × 10^6^ cells from human CRC cell lines were injected subcutaneously into one or both flanks. When the tumor volume reached 500 mm^3^, retransplants were performed as above. All animal work was performed in accordance with a protocol approved by the Local Ethics Committee (decision 59/2013).

### 2.2. Histological Evaluation

Both primary tumors and PDXs were evaluated in regard to several histological features. Tumor grade was assessed based on the ability of cancer tissue to form glands resembling colonic crypts and the percentage of undifferentiated solid epithelial neoplastic cell nests with division for well differentiated (G1), moderately differentiated (G2), and poorly differentiated (G3) colonic adenocarcinoma. The stromal component was measured based on the surface area of fibrotic material between cancerous tissues, given as a percentage of the whole histological image. Within the stromal component, inflammatory infiltrate was assessed as absent, mild, moderate, or intense, with cut-off points at 25% and 50% of the area occupied by inflammatory cells. The characteristics of these infiltrates were also evaluated, and the predominant inflammatory cells were described as mononuclear (lymphocytes and monocytes). In addition, tumor budding was identified as small groups of neoplastic cells (fewer than five) separating from the glands and invading the stroma. The intensity of tumor budding was measured semiquantitatively, as described previously in literature: the areas of most pronounced budding were selected under low power, and then tumor budding foci were counted under a 20× objective lens, with < 5 indicating low-grade and ≥ 5 high-grade budding. Immunohistochemistry staining with human leukocyte antigen was performed on each tissue section to identify cells originating within both the epithelial and stromal components of the tumor. Kidney tissue was used as a negative control.

### 2.3. Next-Generation Sequencing

RNA and DNA were extracted using the RNeasy Mini Kit and QIAamp DNA Mini Kit (Qiagen), respectively. DNA concentration was measured fluorescently using a Qubit instrument (Thermo). DNA was subjected to library preparation for the Ion AmpliSeq Comprehensive Cancer Panel, which allows analysis of the coding regions of 409 oncogenes and tumor suppressor genes. Quality assessment and quantitation of total RNA were performed using Agilent RNA kits on a Bioanalyzer 2100 (Agilent), followed by library preparation with the Ion AmpliSeq Transcriptome Human Gene Expression Panel (Thermo) as described previously [9]. DNA libraries were sequenced on an Ion Proton sequencer.

### 2.4. Variant Calling

Sequencing results were mapped separately to the human (hg19) and mouse (mm10) genomes using TMAP. Two datasets were created using samtools (10): one containing reads that mapped to the human genome with MAPQ ≥ 30 (“relaxed dataset”), and the other containing reads that mapped to the human genome with MAPQ ≥ 30 but did not map to the mouse genome with MAPQ ≥ 17 (“strict dataset”). Variant calls were made with Torrent Variant Caller, using default parameters for somatic variants. Called variants were first filtered with bcftools with the following parameters: DP ≥ 20, QUAL ≥ 20, and GQ > 5 for all variants; FDP > 6, FAO > 2, and STB < 0.9 for SNPs; and FDP > 10, HRUN < 6, and FAO > 4 for indels. The resultant variants were then filtered with fpfilter with default parameters except as follows: min-strandedness = 0.05, max-mapqual-diff = 10, max-readlen-diff = 10, and max-mm-qualsum-diff = 50. Only variants with at least five alternate reads from each strand and at least 10% alternate reads were selected. To exclude any artifact arising from homologous mapping of mouse DNA to the human reference, all variants arising more frequently in the PDX than in the initial samples (N > 3) in the relaxed dataset were excluded. Variants detected in more than 25% of samples in the relaxed or strict dataset were also removed. Finally, “strict” and “relaxed” datasets were merged. Annotation of variants and prediction of their consequences for mature proteins were conducted using Annovar [10], and deleteriousness was assessed using the SIFT [11]. Only variants not present in more than 0.1% of the population (germline), according to the ExAC and 1000 Genomes databases, were considered. Variants previously linked to CRC development were imported from the COSMIC database (version 20170411 [12]).

### 2.5. Expression Analysis

Transcripts were quantified using HTseq-count (version 0.6.0 [13]), run with default options. Differential gene expression was evaluated with DESeq2 [14]. The 20% of genes with the lowest normalized read counts across all samples were discarded before comparisons were made. Gene Ontology (GO) analysis was performed with the clusterProfiler package [15]. For the purpose of GO analysis, the selected gene set consisted of genes with adjusted p values lower than 0.05 according to DESeq2, and the background consisted of all genes taken to pairwise comparisons. All calculations were performed in the R environment [16].

## 3. Results

### 3.1. Xenograft Establishment

Fresh colorectal carcinoma specimens from 37 surgical resections and five human CRC cell lines (Colo-205, HCT-116, HT-29, SW-480, and Caco2) were grafted subcutaneously into the dorsal region of nude mice. Tumor sizes were measured once a week, and tumors were allowed to grow until they reached ~500 mm^3^. The generation harboring the patient- or culture-derived material, termed P0, was subsequently propagated in consecutively numbered generations (P1, P2, P3, and so on). Altogether, 20 of the 37 tumor samples and all given cell lines yielded growing tumors at graft sites. Engraftment rates of PDXs and CLXs were 54% and 100%, respectively. The times between implantation and the development of progressively growing PDXs and CLXs were 50–162 days and 18–202 days, respectively; the median times for development of P0, P1, P2, and P3 xenografts were 105, 51, 52, and 55 days (PDXs) and 35, 33, 26, and 24 days (CLXs), respectively. Portions of each fresh parental tumor and all xenografts were fixed in formalin and paraffin-embedded for pathological assessment, and other portions were cryopreserved for genetic and molecular surveys.

### 3.2. Xenograft Histology

**Figure 1:**
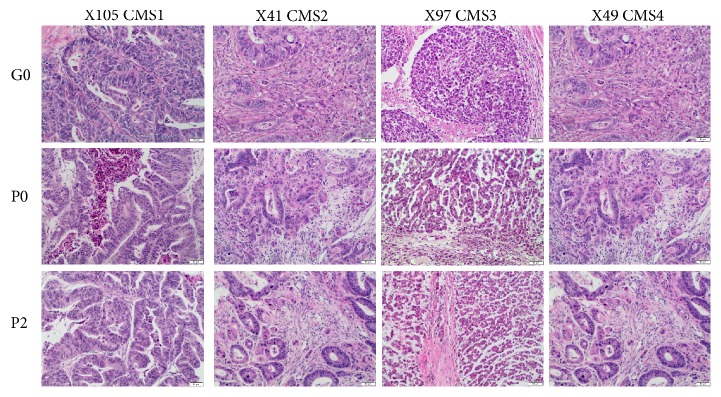
Morphological features observed in xenograft tumors are preserved. Examples show hematoxylin–eosin (H&E) staining of representative primary CRC tumor (GO) and PDX P0 and P2 samples belonging to the one of consensus molecular subtypes (CMS) as determined in transcriptomic survey (10x objective).

As assessed by an experienced pathologist (M.A.), most histological parameters remained stable between the original tumors and engrafted and passage PDXs displayed (Figure 1). Tumor grade did not change except in one case, in which a G2 adenocarcinoma evolved into a mucinous subtype of colonic adenocarcinoma or the tumor grade increased from G2 to G3. Specific analysis of the minor G3 component in tumors confirmed that this feature remained stable in subsequent passages. Similarly, the percentage of the stromal component was nearly identical in the original and transplanted tumors: the variation was less than 10%, near the discrimination limit of the measurement technique. Interestingly, no necrotic areas were observed in engrafted or transplanted tumors unless necrosis was also present in the original tumor. Evaluation of inflammatory infiltrate revealed that the intensity of inflammation remained generally stable, whereas the predominant type of inflammatory cells varied with no obvious pattern. Tumor budding was consistent in terms of presence and intensity in five cases, whereas in another five cases the intensity of budding decreased. Together, these observations confirm that PDXs at early passages (P0–P3) are closely related to clinical cases. Immunohistochemical staining revealed the epithelial component of each tumor, including the foci of tumor budding. In addition, in some engrafted tumors several stromal cells with fibroblast morphology expressed human HLA. In these cases, the number of HLA-positive cells decreased in subsequent passages (Figure S1).

**Figure 2:**
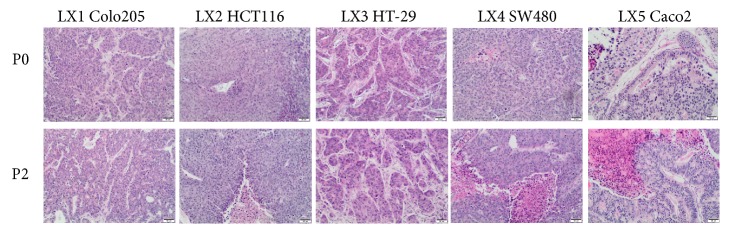
Hematoxylin–eosin staining of CRC cell lines COLO 205, HCT 116, HT29, SW480, Caco-2 (G0), and their second passage in mice (P2) (10x objective).

Among the xenografts derived from human CRC lines, all of the tumors were of G3 histological grade with a small (≤ 15%) stromal component (Figure 2). The stroma in CLXs was less dense and contained some inflammatory cells, mostly on the edges of the specimen. In most cases, the percentage of necrosis was significant at the engraftment site and increased in subsequent passages, reaching 60–85% of the whole tumor area. Due to the high histological grade in all cases, it was not possible to assess tumor budding. The stromal compartment was likely mobilized from elements of murine tissues.

We also assessed 17 surgical specimen tumors from patients in whom part of the tissue was harvested, and the engrafted tumor failed to grow. These tumors were comparable to those previously described, i.e., similar rates of G2 and G3 tumors with similar stromal components. Thirteen cases exhibited tumor budding, and inflammatory infiltrate was present in all tumors.

### 3.3. Single-Nucleotide Variants of Xenografts

**Figure 3:**
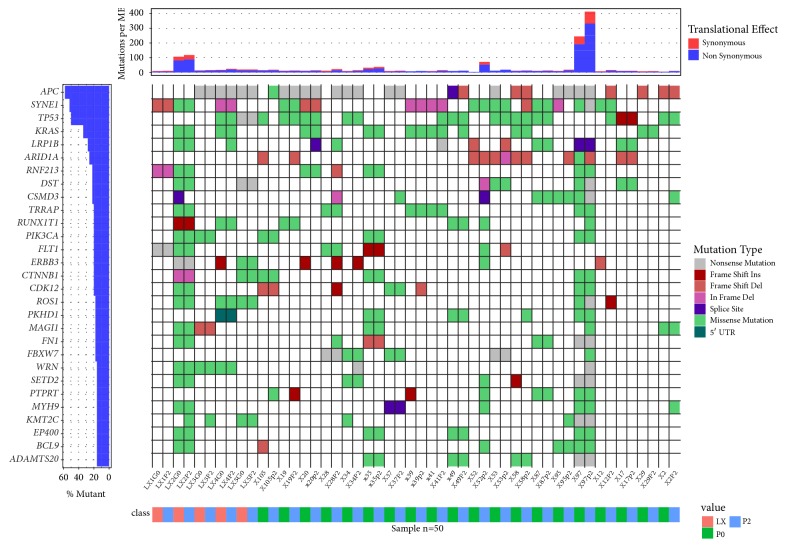
Mutational waterfall plot of genes mutated in > 10% of samples. Mutation frequency, shown in the top panel, is calculated relative to the assayed DNA length (1.29 Mb).

Primary tumors, cell lines, and the corresponding P2 xenografts were surveyed for genetic variants within a panel of 409 cancer-related genes. We identified a total of 2832 single-nucleotide variants (SNVs) and short indels, and average coverage was 1701× (Table S1). Variants were found in 366 genes, with an average of 0.14 variants per gene per sample. Mutations occurred most frequently in* SYNE1* (64% of samples),* TP53* (60%),* APC* (58%),* CSMD3* (52%),* LRP1B* (46%),* PTPRD* (36%),* KRAS* (34%),* MAGI1* (32%), and* RNF213* (30%) (Figure 3). 287 variants, with an average of 0.23 variants per gene per sample, impacted CRC driver genes, as defined by the Cancer Gene Census [17]. Of these, 228 variants were shared, while 48 and 11 were unique for primary tumors or cell line and P2 xenografts, respectively; therefore most of these mutations (79%) were retained in xenografts tissues. For example, KRAS driver mutations were concordant in six primary tumors, PDX P2 pairs with the exception of X49 sample where KRAS p.G12D emerged in PDX only (Figure S3).

**Figure 4:**
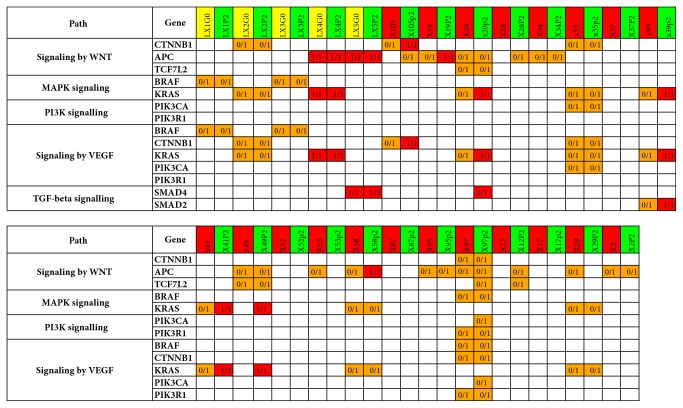
Deleterious variants in genes belonging to selected signaling pathways. Yellow, red, and green column names indicate cell lines, tissue specimens, and PDXs, respectively. Orange and red fields denote heterozygous and homozygous variants, respectively. Please note that heterozygous calls may reflect a mixture of homozygous (i.e., reference and alternative) cells.

We then used the driver mutations as input for annotation using the KEGG signaling pathway database. As shown in Figure 4, the selected signaling pathways that were commonly affected by mutations (Wnt, MAPK, PI3K-Akt, VEGF, and TGF-beta) overlapped extensively between primary tumors or cell lines and the corresponding xenografts. The allele frequency of mutated variants compared with reference variants within these private mutations was usually elevated in xenografts, sometimes reaching 100% (Figure S2). Additionally, the median number of mutations per MB was 10.9 and 14.7 for primary tumors or cell lines and the corresponding xenografts, respectively (Figure S4).

### 3.4. Transcriptome Survey

We analyzed gene expression in all cell lines, P2 CLXs, tumor tissues, and P2 PDXs by RNA-seq. A pairwise comparison revealed that 3262/222 and 918/216 genes were downregulated and upregulated, respectively, in P2 PDX/CLX samples relative to the corresponding source material (Table S2). Because the number of differentially expressed genes was higher in PDX samples, we performed downstream functional analyses for this dataset only. Among the significantly upregulated genes in P2 PDX, GO analysis of Biological Process category revealed overrepresentation of genes involved in cell division and catabolic processes, consistent with the stabilization and accelerated growth of PDXs (Table S3). On the other hand, significantly downregulated transcripts in xenografts were enriched in GO Biological Process terms associated with extracellular matrix organization, immune response, and angiogenesis, in line with the loss of transcripts primarily expressed in stromal cells.

**Figure 5:**
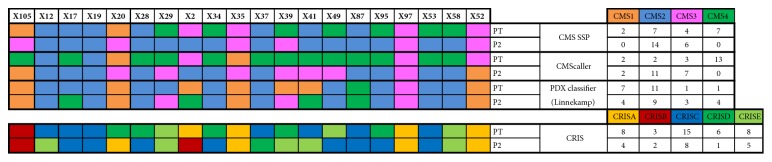
Molecular subtypes of primary tumors (PT) and PDXs derived from them (P2) across four classification systems: the CMS single sample predictor (SSP) [18], CMScaller [20], the CRC intrinsic subtypes (CRIS) classifier [21], and the PDX classifier [19].

To further characterize our set of xenografts at the molecular level, we applied the recently proposed consensus molecular subtype (CMS) classification of CRC tumors, which enables the categorization of most tumors into one of four CRC subtypes [18]. Among primary colon cancers that gave rise to a PDX, the CMS classifier based on the single sample predictor (SSP) assigned two, seven, four, and seven into the CMS1 (MSI-immune), CMS2 (epithelial and canonical), CMS3 (epithelial and metabolic), and CMS4 (mesenchymal) subtypes, respectively (Figure 5). On the other hand, among primary tumors that did not develop into a PDX, the CMS classifier allocated one, four, five, and seven to CMS1, CMS2, CMS3, and CMS4, respectively. CMS frequencies did not differ significantly among primary tumors that did and did not develop into a PDX (noPDX). Interestingly, 324 (133/191 up-/downregulated in noPDX vs. PDX) transcripts were significantly altered between these two types of primary tumors (Table S4). GO analysis of the set of downregulated genes revealed enrichment in Biological Process terms related to ribonucleoprotein complex biogenesis and rRNA processing, in tumors that did not develop into a PDX (Table S5).

The CMS predictor assigned all PDXs to two molecular subtypes, CMS2 or CMS3 (Figure 5). The failure of the CMS classifier to identify CMS1 and CMS4 subtypes has been described previously [19]. To address this deficiency, several alternative classifiers have been recently proposed, including the cancer cell–adapted CMScaller [20], the CRC intrinsic subtypes (CRIS) classifier [21], and the PDX classifier [19]. All of these classifiers aim to enrich molecular classification with cancer cell–intrinsic gene expression signals and ignore gene expression signatures connected to stromal components, which are replaced by their murine counterparts during xenotransplantation [1]. The original CMS classifier, CMScaller, the PDX classifier, and CRIS failed to correctly match 10, 17, 5, and 9 PDXs, respectively, to the molecular subtype of the corresponding primary tumors as determined by the same classifier. Thus, out of the four classifiers employed, the PDX classifier proposed by Linnekamp and colleagues [19] performed the best, matching 15 out of 20 PDXs (75%) to the molecular subtype of their primary tumors. In sum, none of the classifiers tested faithfully recapitulated PDX molecular subtype and therefore must be considered imperfect tools for the molecular classification of xenografts; however, each of them did identify molecular differences between donor tumors/cell lines and the xenografts derived from them.

## 4. Discussion

Chemotherapeutics and targeted therapies are the two main groups of drugs used for antineoplastic treatment. Although most cytotoxic anticancer drugs were discovered through random screening of synthetic compounds and natural products in* in vitro* cytotoxicity assays, targets for therapeutics directed against the specific signaling pathways responsible for cancer growth and maintenance are mostly identified by high-throughput technologies in studies conducted* ex vivo* and* in vivo*. During the early steps of novel therapy development, cell line panels are commonly used as a part of the biology discovery phase, and to screen the activity of new compounds* in vitro*. However, cell lines rarely recapitulate the biology and histology of parental tumors either* in vitro* or* in vivo*, even when reimplanted as CLXs. Instead, PDXs better recapitulate the substantial molecular heterogeneity of human tumors and therefore remain the recommended models for target searching and proof-of-concept studies [22, 23].

Ultimate therapeutic response varies significantly between cancer patients due to the high inter- and intratumor genetic and molecular heterogeneity underlying the primary and secondary mechanisms of resistance. Although tumor classification might increase efficacy and diminish the side effects of new therapies, only RAS mutation status is used routinely as a negative predictive marker to avoid treatment with anti-EGFR agents in patients with metastatic CRC. In turn, mismatch repair status can guide the use of adjuvant CHT in patients with early-stage colon cancer [5]. Since the establishment of PDXs as a basic procedure for evaluating therapeutic responses in preclinical studies, the standardization of PDX models has become particularly important [1]. Although a great deal of effort has been devoted to standardizing preclinical CRC models [24–28], differences still exist in the way assessments are made. This is particularly true of evaluations carried out at the molecular level.

PDXs are established by transferring tumor tissue into immunosuppressed mice, but usually not at the original anatomical site. In addition, loss of human immune signatures and stromal components [20] may limit the value of PDX models for studies of the role of the tumor microenvironment aimed at optimizing cancer treatment. In fact, the original CMS classifier fails to identify the CMS1 (immune) and CMS4 (mesenchymal) groups in cell lines, patient-derived organoids, and PDXs [20]. Furthermore, a selection of preexisting minor clones during PDX development and propagation can substantially change the molecular characteristics of PDXs in comparison with their parental tumors [6–8]. As a consequence, simultaneous molecular classification of both primary tumors and their corresponding PDXs may yield superior animal models for preclinical studies of new anticancer agents.

In this study, we compared histology, genetics, and gene expression profiles between CRC primary tumors and xenografts derived from them, with the goal of determining which elements of the primary tumor tissue remain unchanged and which evolve. Most histological parameters remained stable between the original tumors and engrafted/passaged PDXs. For example, PDX grade, stromal component, intensity of inflammation, and budding were consistent in terms of presence and intensity with those of the donor tumor, indicating that PDXs at early passages are closely related to their parental colon cancers. Although at early passages some engrafted tumors contained stromal cells of fibroblast morphology that expressed human HLA, these cells became less abundant in subsequent passages. All five CLXs exhibited G3 histological grade with a reduced proportion of dense stromal components. Although PDXs contained little or mild tissue necrosis, in CLXs necrosis reached up to 85% of the whole tumor area. The stromal compartment of CLXs was likely murine. In accordance with these histological differences, both the timeframe required for xenograft engraftment and the average time of P1–P3 generation growth were significantly longer for PDXs than for CLXs. Thus, although PDXs are closer representations of the disease than CLXs, the longer timeframe required for engraftment is an obstacle for their use in co-clinical trials, and individual patients with rapidly progressing disease could not benefit from PDX studies [29].

Among 409 cancer-related genes, SNVs were identified in 366. Of these, 287 variants impacted CRC driver genes, with mutations occurring most commonly in* APC*,* KRAS*,* CTNNB1*,* PIK3CA*,* FBXW7*,* UBR5*,* PTPRT*, and* TP53*. Several mutations were comparably deleterious. Of note, the driver mutations that affected CRC signaling pathways were mostly represented by private variants that exhibited almost complete concordance between the primary tumors or cell lines and their respective xenografts, with the fraction of nonreference reads usually increasing in xenografts. Thus, while the mutational profiles reflected significant intertumor differences, the prevalence of mutated variants relative to reference variants was typical for PDXs. Moreover, the donor cell lines and the CLXs derived from them revealed not only virtually identical mutation patterns but also identical ratios between mutated and reference variants. Although our preclinical models likely capture only part of the genetic heterogeneity of CRC donor tumors, these differences might reflect subclonal selection during the process of engrafting or propagation of the growing xenografts. However, we cannot rule out the possibility that the increase in the frequency of mutant variants in xenografts represents DNA extracted mostly from neoplastic cells, whereas the DNA isolated from primary tumors was also derived from noncancerous cells.

Loss of human immune signatures and stromal components and selection of preexisting minor clones during PDX development and propagation represent specific challenges for the processes of transcriptome survey and PDX sample classification by CMS. In the transcriptome survey, we attempted to assign the gene expression of primary tumor/cell lines and their corresponding xenografts to CRC molecular subtypes using four classifiers: the original CMS classifier [18], CMScaller [20], the CRIS classifier [21], and the PDX classifier [19]. None of them can be considered as a gold standard, although each identified molecular differences between donor tumors/cell lines and xenografts. In particular, the original CMS classifier failed to identify CMS1 and CMS4 in PDX samples. On the other hand, the proposed alternative classifiers are likely not completely independent of tumor stroma; filtering of the stromal component, although it reduces the influence of stromal gene expression on final classification, may significantly affect assignment of a PDX to a molecular subtype. For example, surprisingly, CMScaller assigned 13 out of 20 primary tumors to CMS4, whereas according to this classifier this subtype was absent in PDXs. Thus, for accurate standardization of preclinical studies, we need a more robust molecular classifier that is insensitive to differences in stromal components between human cancer tissues and their corresponding xenografts. It should be highlighted that the original CMS classifier and its alternatives were proposed and tested on the transcriptome datasets generated by applying microarray technology to material from primary tumors. It is to be expected that the xenotransplantation process itself interferes with the transcriptome of cancer cells in several ways, including dominant clone selection upon engraftment, limited cross-talk between human and mouse cellular components, and lack of a functional immune system in the host animals [18]. Recent work showed that PDX propagation causes changes in DNA copy number, allowing clones with a minor representation in the xenograft to obtain a fitness advantage over the course of PDX passage [6]. On the other hand, murine stroma cells adapt a human-like metabolome profile in PDXs [30]. Given the divergent and common features of PDX models and primary tumors, an extended PDX-tailored classifier that includes multiple molecular features should be developed and trained on the growing number of thoroughly characterized CRC PDX samples. Ultimately, the purpose of the classification system, like those for primary tumors, is to provide guidance for PDX stratification in co-clinical trials and subtype-based targeted interventions. However, because PDX molecular profiling still has serious limitations, selection of these models for preclinical studies should also take into account more stable parameters, such as the presence of targeted genetic variants, along with evaluation of the response to standard anticancer therapies.

In summary, we have described our experience with CRC xenografts, one of the molecularly best-characterized models of solid tumors. Despite their limitations, the continuously growing collection of CRC PDXs constitutes a valuable tool for preclinical and translational research. The development of new “humanized” preclinical CRC models, with engraftment of human immune systems into immunodeficient mice [31], introduction of human gut microbiota [32], or the* in vivo* reconstruction of the human colon epithelium [33], is a promising approach to filling the translational gap between PDX models and primary CRC tumors.

## Data Availability

Gene expression data has been deposited in Gene Expression Omnibus database, entry GSE112941.

## Supplementary Materials

Supplementary Materials Figure S1: immunohistochemistry of HLA confirms loss of the human component in PDX tissue stroma (10x objective). Figure S2: fraction of reads supporting deleterious variants in TP53 in primary tissue (red columns) and PDXs (green columns). No deleterious variants were found in cell lines. Fields with values larger than 0.66 are marked red. REF: reference variant; ALT: alternative variant. Figure S3: waterfall plot of the most frequently mutated cancer driver genes. Figure S4: number of single nucleotide variants and short indels identified in each sample. Samples LX2 and X97 had more than 100 and 400 variants/MB, respectively, and do not fit the scale (see the first panel in Figure 3). Supplementary Tables 1-5 are deposited in a separate excel file.
