# Supplementary material for: Challenges in Stratifying the Molecular Variability of Patient-Derived Colon Tumor Xenografts
Source: Biomed Res Int. 2018 Dec 19;2018:2954208. doi: 10.1155/2018/2954208 (PMC6313970; doi:10.1155/2018/2954208)

Supplementary figures to manuscript:

Challenges in stratifying the molecular variability of patient-derived colon tumor xenografts

Magdalena Cybulska[1,2#], Tomasz Olesinski[3#], Krzysztof Goryca[1], Katarzyna Paczkowska[1], Malgorzata Statkiewicz[1], Michal Kopczynski[1], Aleksandra Grochowska[1,2], Katarzyna Unrug-Bielawska[1], Anita Tyl-Bielicka[1], Marta Gajewska[1], Andrzej Mroz[3], Michalina Dabrowska[1], Jakub Karczmarski[1], Agnieszka Paziewska[2], Leszek Zając[3], Mariusz Bednarczyk[3], Michal Mikula[1*], Jerzy Ostrowski[1,2,*]

[1] Department of Genetics, Maria Sklodowska-Curie Institute – Oncology Centre; 02-781 Warsaw, Poland

[2] Department of Gastroenterology, Hepatology and Clinical Oncology, Medical Center for Postgraduate Education; 01-813 Warsaw, Poland

[3] Department of Gastroenterological Oncology, Maria Sklodowska-Curie Institute – Oncology Centre; 02-781 Warsaw, Poland

[#] Equal contribution

*Corresponding Author:

Jerzy Ostrowski, MD, PhD; Maria Sklodowska-Curie Institute – Oncology Centre, Roentgena 5, 02-781 Warsaw, Poland, Tel: +48 225462575, Fax: +48 225462449, E-mail: jostrow@warman.com.pl

Michal Mikula, PhD; Maria Sklodowska-Curie Institute – Oncology Centre, Roentgena 5, 02-781 Warsaw, Poland, Tel: +48 225462655, Fax: +48 225462449, E-mail: mikula.michal@coi.pl

Figure S1. Immunohistochemistry of HLA confirms loss of the human component in PDX tissue stroma (10x objective).

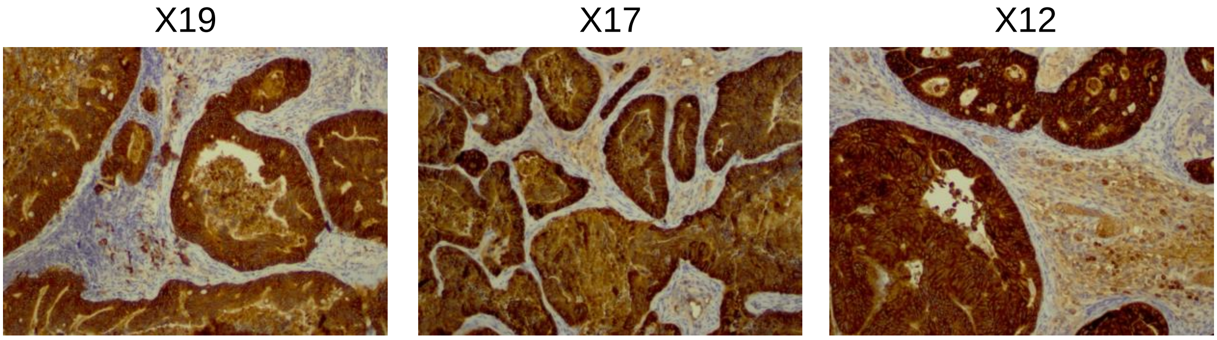

Figure S2. Fraction of reads supporting deleterious variants in *TP53* in primary tissue (red columns) and PDXs (green columns). No deleterious variants were found in cell lines. Fields with values larger than 0.66 are marked red. REF - reference variant, ALT - alternative variant.

Genomic position	REF	ALT	X105	X105p2	X19	X19P2	X20	x20p2	X28	X28P2	X34	X34P2	x35	x35p2	X37	X37P2	x39	x39p2	x41	X41P2	x49	X49P2
7577538	C	T																			0.13	1
7577547	C	T																		1		
7577570	C	A									0.49	1										
7578265	A	G	0.21																			
7578281	G	T			0.56	0.99																
7578404	A	G					0.43	1														

Genomic position	REF	ALT	X52	X52p2	X53	X53p2	X58	X58p2	X87	X87p2	X95	X95p2	X97	X97p2	X12	X12P2	X17	X17p2	X29	X29P2	X2	X2P2
7576896	T	TG															0.47	0.97				
7577094	G	A					0.6	1														
7577570	C	T														1						
7578406	C	T			0.68	0.99			0.42	1											0.12	1

Figure S3. Waterfall plot of the most frequently mutated cancer driver genes.

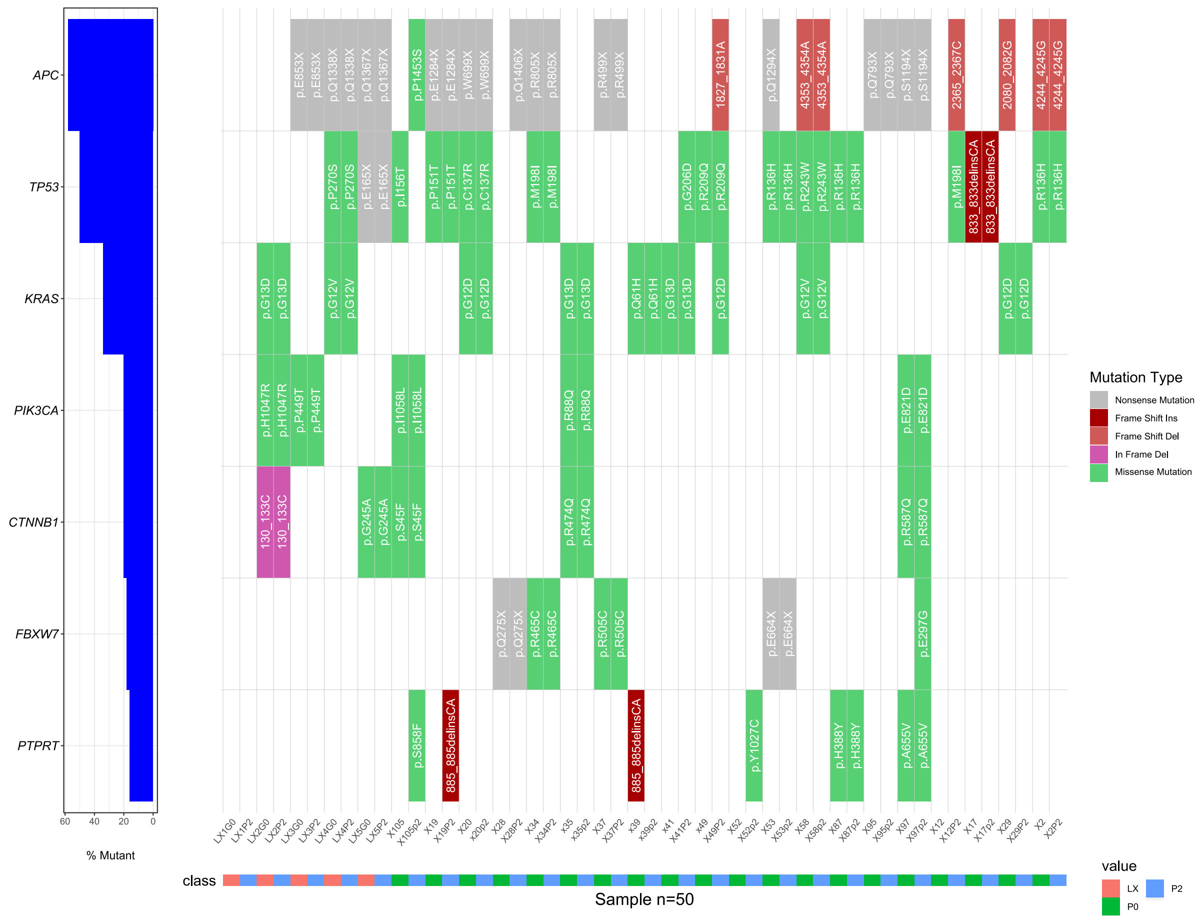

Figure S4. Number of single nucleotide variants and short indels identified in each sample. Samples LX2 and X97 had more than 100 and 400 variants/MB, respectively, and don't fit the scale (see the first panel in Figure 3).

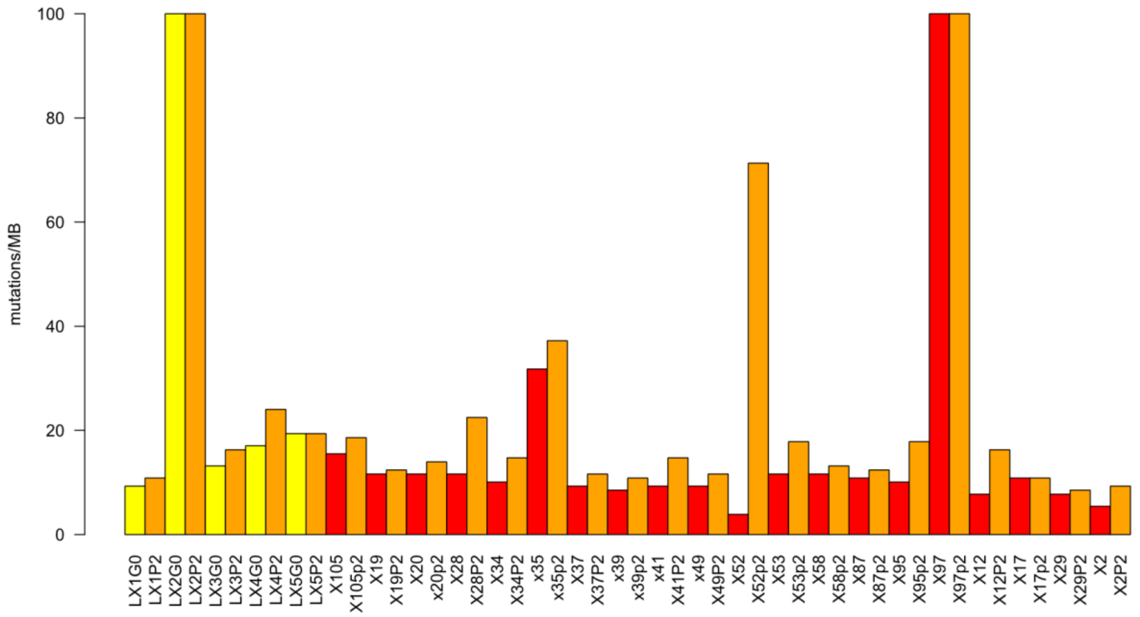

Supplement: Supplementary Materials — Figure S1: immunohistochemistry of HLA confirms loss of the human component in PDX tissue stroma (10x objective). Figure S2: fraction of reads supporting deleterious variants in TP53 in primary tissue (red columns) and PDXs (green columns). No deleterious variants were found in cell lines. Fields with values larger than 0.66 are marked red. REF: reference variant; ALT: alternative variant. Figure S3: waterfall plot of the most frequently mutated cancer driver genes. Figure S4: number of single nucleotide variants and short indels identified in each sample. Samples LX2 and X97 had more than 100 and 400 variants/MB, respectively, and do not fit the scale (see the first panel in Figure 3). Supplementary Tables 1-5 are deposited in a separate excel file. [file 2954208.f1.zip › 2954208.f1/Supplementary figures 1-4_BMRI_2603419.pdf]
